# The systemic immune-inflammation index is an independent predictor of survival for metastatic colorectal cancer and its association with the lymphocytic response to the tumor

**DOI:** 10.1186/s12967-018-1638-9

**Published:** 2018-10-04

**Authors:** Qian-Kun Xie, Ping Chen, Wan-Ming Hu, Peng Sun, Wen-Zhuo He, Chang Jiang, Peng-Fei Kong, Shou-Sheng Liu, Hai-Tian Chen, Yuan-Zhong Yang, Dan Wang, Lin Yang, Liang-Ping Xia

**Affiliations:** 1 VIP Region, State Key Laboratory of Oncology in South China, Collaborative Innovation Center for Cancer Medicine, Sun Yat-Sen University Cancer Center, 651 Dongfeng East Road, Guangzhou, 510060 Guangdong People’s Republic of China; 2 Department of Pathology, State Key Laboratory of Oncology in South China, Collaborative Innovation Center for Cancer Medicine, Sun Yat-Sen University Cancer Center, Guangzhou, 510060 Guangdong People’s Republic of China; 3 Department of Pathology, School of Basic Medical Sciences, Southern Medical University, Guangzhou, 510515 China; 4 Department of Pathology, Nanfang Hospital, Southern Medical University, Guangzhou, 510515 China; 5 Zhongshan School of Medicine, Sun Yat-Sen University, Guangdong, 510060 People’s Republic of China; 6 State Key Laboratory of Oncology in South China, Collaborative Innovation Center for Cancer Medicine, Sun Yat-Sen University Cancer Center, Guangzhou, 510060 Guangdong People’s Republic of China

**Keywords:** Metastatic colorectal cancer, Systemic immune-inflammation index, Immunity, Survival

## Abstract

**Background:**

Systemic inflammation and immune dysfunction has been proved to be significantly associated with cancer progression and metastasis in many cancer types, including colorectal cancer. We examined the prognostic significance of the systemic immune-inflammation index (SII) in patients with metastatic colorectal cancer (mCRC) and the relationship between the lymphocytic response to the tumor and this index.

**Methods:**

This retrospective study evaluated 240 consecutive patients with newly diagnosed stage IV mCRC who underwent surgical resection. The SII values were calculated based on preoperative laboratory data regarding platelet, neutrophil, and lymphocyte counts. Tumor-infiltrating lymphocytes were evaluated using the surgical specimens. The overall survival and their 95% confidence interval (95% CI) were estimated by regression analyses and the Kaplan–Meier method.

**Results:**

After a mean follow-up of 26.7 (1.1–92.4) months, 146 patients (60.8%) died. In the univariate analysis, a high SII was significantly associated with poor overall survival (*P *= 0.009). The multivariable analysis also confirmed that a high SII was independently associated with poor overall survival (hazard ratio: 1.462, 95% confidence interval 1.049–2.038, *P *= 0.025). The SII value was significantly correlated with the TILs value at the tumor’s center (*P *= 0.04), but not at the invasive margin (*P *= 0.39). When we evaluated overall survival for groupings of the tumor-infiltrating lymphocytes and SII values, we identified three distinct prognostic groups. The group with low tumor-infiltrating lymphocyte values and high SII values had the worst prognosis.

**Conclusions:**

A high SII value independently predicts poor clinical outcomes among patients with mCRC. In addition, combining the lymphocytic response to the tumor and SII could further enhance prognostication for mCRC.

**Electronic supplementary material:**

The online version of this article (10.1186/s12967-018-1638-9) contains supplementary material, which is available to authorized users.

## Background

Colorectal cancer (CRC) is a leading cause of morbidity and mortality worldwide [1], and approximately 25% of patients with CRC have distant metastasis at the initial diagnosis [2]. In the era of cytotoxic drug combinations and molecular targeting agents, the integration of surgery and effective systemic chemotherapy has emerged as a new strategy for prolonging survival of patients with metastatic colorectal cancer (mCRC) [3–7]. However, in most cases the disease is not curable. This leads to further exploration in an effort to understand and improve treatment failure in this subset of mCRC patients.

With the success of the check-point inhibitors in a wide variety of tumor in recent years, the host immune response, notably an enhanced lymphocytic reaction, has become a recent focus of investigation [8–11]. Preexisting cytotoxic T lymphocyte cells in the tumor microenvironment can attack cancer cells by recognizing abnormally expressed neoantigens, and were required for tumor regression after immune checkpoint blockade [12]. Currently, tumor immune response with respect to lymphocytic infiltrations can be assessed in hematoxylin–eosin (H&E)-stained sections basing on the morphology characteristics of cells, which is a first pragmatic and cost-effective approach. Numerous studies confirmed the prognostic value of tumour infiltrating lymphocytes (TILs) in various types of malignancies in recent years [13–16]. Furthermore, the TILs have shown a significant prognostic power in our series of mCRC patients [17].

Inflammation has been recognized as a mechanism of immunoresistance in tumors, promoting cancer development and progression [18]. Via a complete blood count, physician can easily identify immune-inflammatory elements (neutrophils, lymphocytes and platelets), which might shed light on the inflammatory tumour microenvironment [19, 20]. Hu et al. [21] were the first to describe the systemic immune-inflammation index (SII), which is based on neutrophil, lymphocyte, and platelet counts. Subsequent research has indicated that the SII has greater prognostic value for malignant tumors than single-parameter markers such as the neutrophil-to-lymphocyte ratio (NLR), or the platelet-to-lymphocyte ratio (PLR) [22–29]. SII has been preliminarily investigated in CRC patients, Chen et al. was the first to establish the advantage prognostic value of SII than NLR and PLR in patients with CRC after radical surgery [30]. Passardi et al. and Yang et al. also confirmed the prognostic value of SII, however, SII didn’t show advantage than PLR and NLR [31, 32], and not as NLR able to predict the efficacy of bevacizumab in mCRC [32]. However, these studies [30–32] were restricted by the limited information on pathologic features and treatment regimen. In mCRC, the factors such as metastasectomy, adjuvant chemotherapy, and metastasis sites involved may confound each other in survival analysis. Thus, the independent contribution of SII to survival in the context of established prognostic factors remain to be determined in mCRC. To date the existing studies have focused on local lymphocytic reaction or systemic inflammatory responses in isolation, it is of also interest that the relationship between local immune status and the systemic environment in mCRC patients. Therefore, the present study evaluated the prognostic value of the SII in mCRC, whether the SII was correlated with TILs, and whether these factors could be combined to better predict overall survival.

## Methods

### Study population

This retrospective study evaluated 240 consecutive patients with newly diagnosed stage IV CRC who underwent primary tumor resection at our institution during 2009–2014. The eligibility criteria were pathologically confirmed CRC, synchronous distant metastasis at the time of the diagnosis, and available data regarding the preoperative complete blood count and follow-up results. The exclusion criteria were inflammatory bowel disease-related CRC, known hereditary CRC syndrome, preoperative chemoradiotherapy, and a history of other malignancies within the preceding 5 years. The study’s retrospective protocol was approved by the Ethics Committee of Sun Yat-Sen University Cancer Center, and all patients had provided written informed consent for their data and surgical specimens to be used for research purposes.

The preoperative complete blood count nearest to the time of the operation was used to calculate SII as (platelet count) × (the neutrophil-to-lymphocyte ratio) [21]. The median SII value was used to dichotomize the patients as having high or low SII values. The OS interval was calculated as the time from the surgery until the first instance of cancer-related death or loss to follow-up. Patients who died because of non-CRC causes were censored at the time of their death.

### Tumor-infiltrating lymphocytes

Hematoxylin and eosin-stained sections from primary tumor specimens were retrieved for all patients. Counts for TILs were performed at the center of the tumor and at the invasive margin, which was defined as the interface between the tumor’s invading edge and the host stroma. The density of TILs was graded as 0 (absent), 1+ (mild), 2+ (moderate), or 3+ (marked), based on previous reports [16, 33]. For the present study, a low TILs score was defined a scores of 0–1 and a high score was defined as scores of 2–3. Where there was disagreement regarding TIL category between pathologists, joint reevaluation was performed to arrive at a consensus.

### Statistical analysis

The Mann–Whitney U test was used to analyze differences in the SII distributions between the patient groups. Kaplan–Meier analyses with the log-rank test were performed to compare survival outcomes. Significant baseline characteristics were used for propensity score analysis. Multivariate analysis using a Cox proportional hazards regression model was performed based on variables with a *P*-value of < 0.05 from the univariate analyses. All statistical tests were two-sided and differences were considered significant at *P *< 0.05. All analyses were performed using IBM SPSS software (version 22.0; IBM Corp., Armonk, NY).

## Results

The baseline characteristics of the 240 patients are summarized in Table 1. The median patient age was 59 years (range 18–90 years) and 157 patients (65.4%) were male. The primary tumors were located in the colon (191 patients, 79.6%) or the rectum (45 patients, 18.8%), with 179 patients having node-positive disease (74.6%) and 57 patients having poor differentiation (23.8%). Most patients (63.8%) had metastases in a single organ, although 87 patients (36.3%) had metastatic sites spread over multiple organs. Palliative resection of the primary cancer was performed for 194 patients (80.8%) and 46 of these patients also underwent metastasectomy. Postoperative chemotherapy was performed for 77.1% of these patients.

**Table 1:** Baseline characteristics

	*N *= 240
Age (years)	
< 65	179 (74.6)
≥ 65	61 (25.4)
Sex, no. (%)	
Male	157 (65.4)
Female	83 (34.6)
Histological grade (%)	
Mod/well differentiated	156 (65.0)
Poorly differentiated	57 (23.8)
Other or missing	27 (11.3)
Primary site (%)	
Colon	191 (79.6)
Rectum	45 (18.8)
Others	4 (1.7)
T-stage (depth of invasion) (%)	
T1–3	144 (60.0)
T4	93 (38.8)
Tx	3 (1.3)
N-stage (lymphatic invasion) (%)	
N0	58 (24.2)
N+	179 (74.6)
Nx	3 (1.3)
MMR status (%)	
dMMR	12 (5.0)
pMMR	228 (95.0)
Metastasectomy (%)	
+	46 (19.2)
−	194 (80.8)
No. of metastatic organs (%)	
Single	153 (63.8)
Multiple	87 (36.3)
Adjuvant chemotherapy (%)	
Negative	55 (22.9)
Positive	185 (77.1)
Metastatic sites (%)	
Liver	148 (61.7)
Extrahepatic disease	92 (38.3)

*MMP* mismatch repair, *dMMR* mismatch repair-deficient, *pMMR* mismatch repair-proficient

**Fig. 1:**
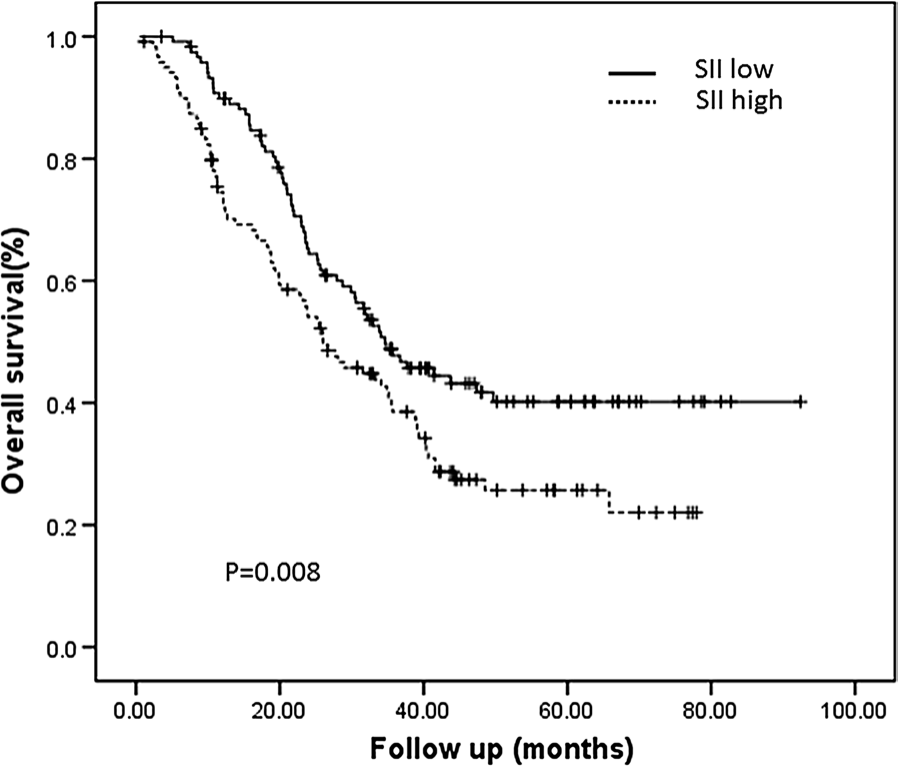
Prognostic value of the systemic immune-inflammation index. Estimated Kaplan–Meier curves for overall survival grouped according to systemic immune-inflammation index (SII) level; in all patients with mCRC (n = 240), hazard ratio = 1.548 95% CI 1.116–2.146, *P* = 0.008; low SII < 649.45; high SII ≥ 649.45

The median SII value was 649.45. Table 2 shows that a high SII value was significantly associated with multiple metastatic sites (*P *= 0.017) and marginally associated with a primary tumor in the rectum (*P* = 0.071). No other associations were observed between the SII and the other clinicopathological factors. Relative to patients with a low SII value, patients with a high SII experienced significantly shorter OS (hazard ratio [HR]: 1.548, 95% confidence interval [CI] 1.116–2.146; log-rank *P *= 0.008, Fig. 1). In the univariate analyses, survival was associated with age, primary site, T-stage, lymph node status, number of metastatic organs, metastasectomy, and adjuvant therapy (Table 3). To eliminate inherent biases, significant factors (age, primary site, T-stage, lymph node status, number of metastatic organs, metastasectomy, and adjuvant therapy) were also used for propensity score analysis (Additional file 1: Table S1). As expected, the raw and normalized results were consistent (*P *= 0.022; Additional file 2: Figure S1). In the multivariate analysis, which was adjusted for those risk factors, a high SII still independently predicted poor OS (HR: 1.462, 95% CI 1.049–2.038; log-rank *P *= 0.025).

**Table 2:** Associations between clinicopathologic variables and SII

Characteristics	Mean	s.e.m.	Median	*P*-value
Age (years)				
< 65	857.17	50.70	637.76	0.36
≥ 65	866.76	81.21	697.67	
Gender				
Male	898.28	56.40	650.76	0.35
Female	786.47	63.49	637.76	
Histologic grade				
Mod/well differentiated	862.53	49.40	699.31	0.27
Poorly differentiated	818.38	87.84	585.0	
Primary site				
Colon	884.17	47.35	697.67	0.07
Rectum	737.66	99.95	595.71	
T-stage				
T1–3	897.49	60.33	675.11	0.55
T4	784.97	58.11	613.79	
LN status				
pN0	933.26	94.50	694.81	0.28
pN+	827.44	48.37	637.94	
MMR status				
dMMR	897.14	195.05	747.13	0.71
pMMR	857.63	44.17	648.99	
Metastatic sites				
Liver	877.09	53.67	668.48	0.43
Extrahepatic disease	831.49	71.88	639.15	
No. of metastatic organs				
Single	797.31	51.82	607.71	0.02
Multiple	969.17	74.88	775.77	

*SII* systemic immune-inflammation index, LN lymph node

**Table 3:** Association of SII with prognosis (overall survival) in the whole study population

	Univariate analysis		Multivariate analysis	
	HR (95% CI)	*P* value	HR (95% CI)	*P* value
Age (years)				
< 65	–	0.003	–	0.018
≥ 65	1.699 (1.197–2.410)		1.540 (1.077–2.202)	
Gender				
Male	–	0.484		
Female	1.128 (0.805–1.579)			
Histologic grade				
Mod/well differentiated	–	0.245		
Poorly differentiated	1.147 (0.910–1.446)			
Primary site				
Colon	–	0.025	–	0.078
Rectum	0.634 (0.426–0.944)		0.685 (0.450–1.043)	
T-stage				
T1–3	–	0.005	–	0.002
T4	1.579 (1.149–2.169)		1.669 (1.203–2.316)	
LN status				
pN0	–	0.006	–	0.028
pN+	1.762 (1.178–2.635)		1.571 (1.049–2.352)	
MMR status				
dMMR	–	0.054		
pMMR	2.415 (0.986–5.916)			
Adjuvant chemotherapy				
Negative	–	0.015	–	0.011
Positive	0.625 (0.429–0.912)		0.602 (0.406–0.892)	
Metastasectomy				
−	–	< 0.001	–	0.004
+	0.380 (0.231–0.624)		0.479 (0.289–0.795)	
Metastatic sites				
Liver	–	0.705		
Extrahepatic disease	0.937 (0.670–1.312)			
No. of metastatic organs				
Single	–	0.008	–	0.010
Multiple	1.350 (1.080–1.688)		1.361 (1.076–1.721)	
SII				
Low	–	0.009	–	0.025
High	1.548 (1.116–2.146)		1.462 (1.049–2.038)	

*SII* systemic immune-inflammation index, *CI* confidence interval, *HR* hazard ratio, *LN* lymph node

The relationship between the SII and TILs values is shown in Table 4. A low TILs value in the tumor’s center was associated with a high pre-operative SII value (P = 0.041). No significant association was observed between the SII value and the TILs value at the invasive margin.

**Table 4:** Associations between inflammation markers and immune cell density in the tumor microenvironment

Lymphocytes	*N* (%)			
	Mean	s.e.m.	Median	*P*-value
Central region				0.04
Low grade	913.41	55.12	697.66	
High grade	749.95	65.37	592.43	
Invasive margin				0.39
Low grade	862.19	45.49	649.45	
High grade	845.45	126.12	655.72	

*SII* systemic immune-inflammation index

**Fig. 2:**
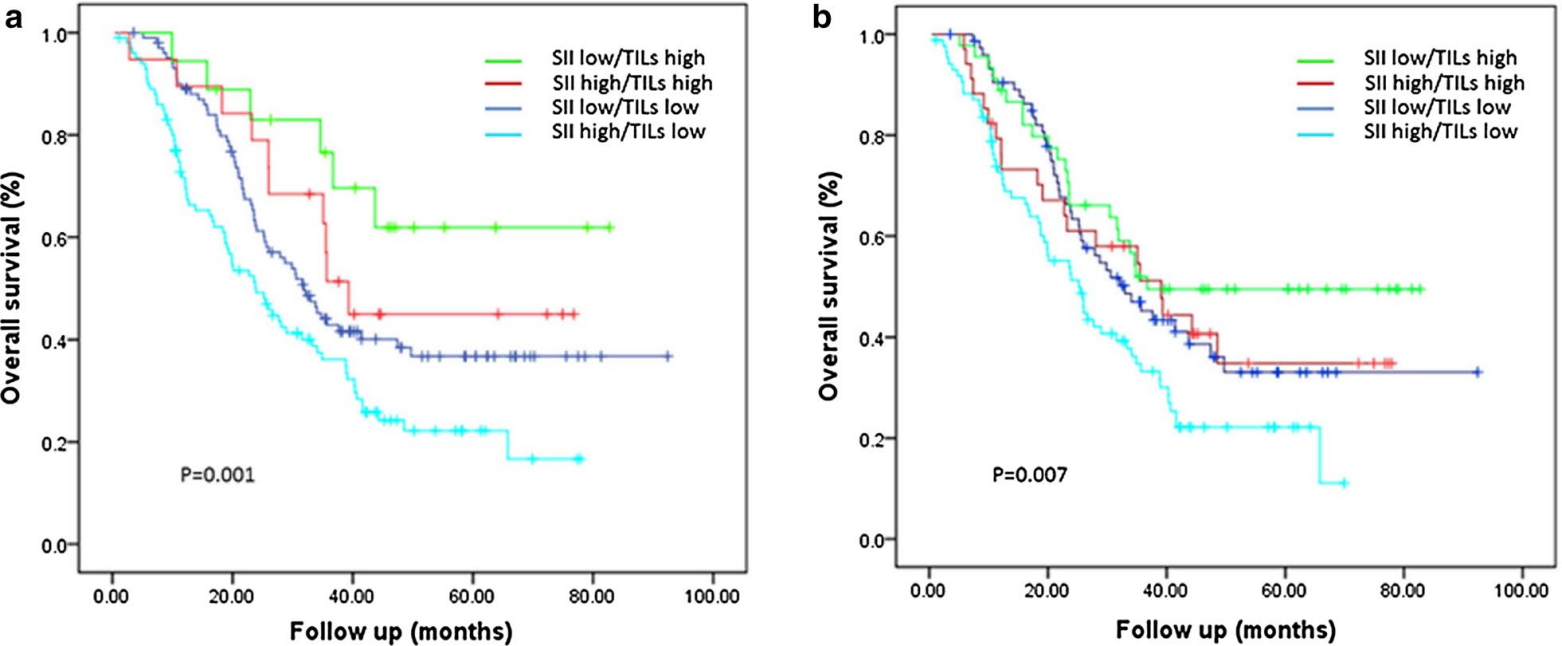
The combined prognostic role of the systemic immune-inflammation index and tumor-infiltrating lymphocytes. Estimated Kaplan–Meier curves for overall survival grouped according systemic immune-inflammation index (SII) and tumor-infiltrating lymphocytes (TILs) category. **a** Patients grouped according to TILs level at the invasive margin and SII; The 5-year overall survival were: low SII and high TILs: 60%; high SII and high TILs: 44%; low SII and low TILs: 37%; high SII and low TILs: 20%. **b** Patients grouped according to TILs level in the tumor’s central region and SII; low SII and high TILs: 49%; high SII and high TILs: 33%; low SII and low TILs: 33%; high SII and low TILs: 21%

Survival outcomes were also evaluated according to combinations of the TILs and SII values, which identified three prognostic groups. Patients with a high TILs value at the invasive margin and a low SII value had the most favorable prognosis, while patients with a low TILs value at the invasive margin and a high SII value had the poorest prognosis (HR: 0.578, 95% CI 0.438–0.763; *P *< 0.001). Patients with either low SII and low TILs values, or with high SII and high TILs values, had similar intermediate prognoses (Fig. 2a). A similar trend was observed when we evaluated the combination of the SII value and TILs value in the tumor’s center (HR: 0.668, 95% CI 0.528–0.846; P = 0.001, Fig. 2b).

## Discussion

To the best of our knowledge, this is the first study to evaluate the clinical relevance of the SII among patients with stage IV CRC and also the first study to evaluate the relationship between the SII and the lymphocytic response to the tumor based on the TILs value. Our results indicate that the preoperative SII value predicted prognosis among our patients, and that the SII value was significantly correlated with the TILs value in the tumor’s center, but not at the invasive margin. Moreover, the combination of the SII and TILs values provided a useful tool for predicting mCRC survival outcomes. These findings suggest that immune and inflammation processes play significant roles in the progression of mCRC.

Elevated levels of inflammatory markers are often associated with more advanced disease, which may be related to a greater tumor burden and/or on-going chronic inflammatory processes [34]. The present study revealed that the SII value was significantly associated with multiple metastatic sites, which agrees with the findings for germ-cell tumors and suggests that systemic inflammation may reflect a tumor’s invasive characteristics [35]. However, the SII value was not associated with other clinicopathological factors, such as T stage and lymph node status. Our further investigation did not find any association between the SII value and the TILs value at the invasive margin, but we observed significant correlation between SII and the presence of TILs in the tumor microenvironment, suggesting the interactions between pro-inflammatory environment and antitumor immunity intratumorally. Thus, systemic inflammation as expressed by SII may be linked to both the tumor and the tumor microenvironment, although the related mechanisms remain incompletely understood.

There is increasing evidence that inflammatory markers can help predict clinical outcomes for various cancers [26, 27, 36–39]. The present study is the first to evaluate the prognostic value of the SII in mCRC and confirmed that high SII values were associated with significantly poorer OS. The prognostic value of this index is likely related to the function of peripheral neutrophils, lymphocytes, and platelets, which are used to determine the SII values. In this context, recent evidence suggests that neutrophils and platelets can promote cancer cell proliferation, invasion, immune evasion, and metastasis via multiple mechanisms [40, 41]. Lymphopenia is especially common in advanced cancer, as observed in the present study, and reflects an inefficient immune system that may produce a favorable microenvironment for the spread of tumor cells [42].

Immune mechanisms have been associated with tumor progression [18]. TILs in the primary tumor predicts prognosis in mCRC were also previously reported in our works [17]. Recognizing the significant role of inflammation and the immune system in the antitumor response and cancer development, it is of interest to define whether systemic inflammation and lymphocytic response could be combined to better predict survival outcomes. We evaluated various combinations of the SII and TILs values, which revealed three prognostic groups. The first group had high TILs and low SII values and experienced favorable prognosis. The second group had low TILs and high SII values and experienced unfavorable prognosis. The third group had either high or low values for both TILs and SII, and experienced similar intermediate prognosis. Thus, it appears that the combination of SII and TILs provides additional prognostic value among cases of mCRC. We speculate that a TILs-related excellent prognosis might be counteracted in a pro-inflammatory environment, although further research is needed to evaluate this possibility and elucidate the underlying mechanism.

Our study has several limitations. First, the retrospective nature limits the availability of blood count information at uniform time points before the operation. Second, the TILs density in full-face stained sections is a relatively crude marker for the antitumor immune response, as it does not differentiate between specific subpopulations of immune cells. Nevertheless, this measure is simple, readily available, and does not require additional immunohistochemical staining, which makes it both practical and cost-effective for clinical use.

## Conclusions

This is the first report to demonstrate that the SII has prognostic value among patients with mCRC, and that the combination of the SII and TILs values provided added prognostic value in this setting. Properly designed prospective studies are needed to further explore these interesting findings.

## Additional files

**Additional file 1: Table S1.** Associations between significant factors and SII after use of PS weighting.

**Additional file 2: Figure S1.** Prognostic value of the systemic immune-inflammation index after use of PS weighting. Estimated Kaplan–Meier curves for overall survival grouped according to systemic immune-inflammation index (SII) level after use of PS weighting. The log-rank test was used to compare the curves.

## Abbreviations

SII: systemic immune-inflammation index
mCRC: metastatic colorectal cancer
TILs: tumour infiltrating lymphocytes
HR: hazard ratio
CI: confidence interval
