# Supplementary material for: The systemic immune-inflammation index is an independent predictor of survival for metastatic colorectal cancer and its association with the lymphocytic response to the tumor
Source: J Transl Med. 2018 Oct 4;16:273. doi: 10.1186/s12967-018-1638-9 (PMC6172841; doi:10.1186/s12967-018-1638-9)
Supplement: Supplementary file 1 — Additional file 1: Table S1. Associations between significant factors and SII after use of PS weighting. [file 12967_2018_1638_MOESM1_ESM.docx]

*Additional file 1: Table S1:*Associations between significant factors and SII after use of PS weighting

| *N* (%) | | | |
| --- | --- | --- | --- |
| Characteristics | Low SII | High SII | *P*-value |
| Age(years) |  |  |  |
| <65 | 89 | 86 | 0.643 |
| ≥65 | 26 | 29 |  |
| Primary site |  |  |  |
| Colon | 90 | 95 | 0.320 |
| Rectum | 24 | 18 |  |
| T-stage |  |  |  |
| T1-3 | 67 | 72 | 0.352 |
| T4 | 48 | 40 |  |
| LN status |  |  |  |
| pN0 | 26 | 30 | 0.465 |
| pN+ | 89 | 82 |  |
| Adjuvant chemotherapy |  |  |  |
| Negative | 25 | 28 | 0.639 |
| Positive | 90 | 87 |  |
| Metastasectomy |  |  |  |
| - | 94 | 95 | 0.863 |
| + | 21 | 20 |  |
| No. of metastatic organs |  |  |  |
| Single | 80 | 70 | 0.166 |
| Multiple | 35 | 45 |  |

Abbreviations: SII, systemic immune-inflammation index ; LN, lymph node
